# Characteristics of Prognostic Programmed Cell Death–Related Long Noncoding RNAs Associated With Immune Infiltration and Therapeutic Responses to Colon Cancer

**DOI:** 10.3389/fimmu.2022.828243

**Published:** 2022-05-31

**Authors:** Yan Chen, Yue Zhang, Jiayi Lu, Zhongchen Liu, Shasha Zhao, Mengmei Zhang, Mingzhi Lu, Wen Xu, Fenyong Sun, Qi Wu, Qi Zhong, Zhongqi Cui

**Affiliations:** ^1^ Department of Clinical Laboratory, Shanghai Tenth People’s Hospital, Tongji University, Shanghai, China; ^2^ Department of Central Laboratory, Clinical Medicine Scientific and Technical Innovation Park, Shanghai Tenth People’s Hospital, Shanghai, China; ^3^ Department of Gastrointestinal Surgery, Shanghai Tenth People’s Hospital Affiliated to TongJi University, Shanghai, China; ^4^ Shanghai Clinical College, Anhui Medical University, Hefei, China

**Keywords:** programmed cell death, colon cancer, lncRNA, immune infiltration, TMB, therapeutic response

## Abstract

Programmed cell death (PCD) plays an important role in the onset and progression of various cancers. The molecular events surrounding the occurrence of abnormally expressed long noncoding RNAs (lncRNAs) leading to colon cancer (CC) have become a focus. We comprehensively evaluated the roles of PCD-related lncRNAs in the clinical management of CC and their immune responses. Therefore, we screened 41 prognostic PCD-related lncRNAs in The Cancer Genome Atlas database using co-expression analysis and assigned patients to groups according to the results of cluster analysis. The immune response and functions of cluster 2 were substantially suppressed, which might explain the poor prognosis in this group. A prognostic model comprising eight PCD-related lncRNAs was developed, and its effectiveness was verified using an external database. High-and low-risk groups had different epigenetic modifications and changes in immune cell infiltration. Patients in the high-risk group were resistant to immunotherapy and various chemotherapeutic drugs. Studies *in vitro* and *in vivo* further confirmed a carcinogenic role of the lncRNA U62317.4. Our findings of the prognostic value of PCD-related lncRNAs revealed their important roles in immune response disorders, thus providing valuable insights into the clinical management and molecular mechanisms of CC.

## Introduction

Among gastrointestinal malignancies, colon cancer (CC) is the second most common cause of tumor-related deaths worldwide (1). An increasing incidence (2) and high mortality rates has led to CC becoming a serious threat to human health (3). Research data show that the 5-year survival rate of early colon cancer (85%-90%) is much higher than that of advanced colon cancer (<14%) (4, 5), however, the clinical management of colon cancer has not yet achieved satisfactory results. Therefore, it is urgent to develop a new biological Markers for prognostic assessment and clinical management of patients.

Cancer cells utilize the immunosuppressive network and create a tumor microenvironment (TME) that allows them to evade host immune surveillance and the potent antitumor activity of immune cells. Numerous cell death processes are initiated in the TME as a result of normal biological responses, external stimuli, or responses to treatment. Apoptosis, pyroptosis, and ferroptosis play important roles in immunogenic cell death (6, 7). Apoptosis is the traditional mode of programmed cell death (PCD) that consists primarily of a mitochondria-mediated intrinsic pathway and an extrinsic pathway involving death receptors (8). Its characteristic features are cell shrinkage, chromatin agglutination, and apoptotic body formation (9). Pyroptosis is a recently-discovered, iron-dependent, novel mode of PCD characterized by continuous cell expansion until the cell membrane ruptures, releasing cellular contents and activating an intense inflammatory response. Pyroptosis is an important part of the natural immune response that plays important roles in combating infections and malignancies (10). Ferroptosis is a novel iron-dependent mode of PCD. The main mechanism is the catalyzed lipid peroxidation of abundant unsaturated fatty acids on the cell membrane by ferrous ions or ester oxygenases, which induces cell death (11). The resulting immune response to changes in the death activities of TME components can affect tumor development and the efficiency of anticancer therapeutics (12, 13).

Long noncoding RNAs (lncRNAs) comprise > 200 nucleotides and they influence many disease processes such as tumors (14, 15) and regulate PCD. For example, the lncRNA HOTAIR-miR-20a-5p-HMGA2 axis plays an important role in the growth, migration, invasion, and apoptosis of breast cancer cells (16). The lncRNA, HOTTIP, inhibits cell pyrolysis in ovarian cancer by targeting the microRNA (miR)-148a-3p/AKT2 axis (17). Furthermore, LINC00336 inhibits ferroptosis during carcinogenesis by interacting with Embryonic Lethal, Abnormal Vision, Drosophila, Homolog-Like 1 to reduce intracellular levels of iron and lipid reactive oxygen species (18). However, only a few biological functions of lncRNAs have been conclusively verified. The clinical significance of most lncRNAs, especially those associated with PCD, remains unclear. Therefore, exploring and understanding the role(s) of PCD-related lncRNAs in CC are important to improve the diagnosis and prognosis of patients.

We analyzed the roles of PCD-related lncRNAs in the prognosis of CC patients and their correlations with clinicopathological characteristics; we then verified the accuracy of our prognostic model using external databases. We also explored the correlations between PCD-related lncRNAs and the TME, immune checkpoint genes, tumor mutation burden (TMB), immunotherapy, and chemotherapeutic drug sensitivity. Thereafter, we analyzed the biological functions of lncRNA U62317.4 in CC *in vitro* and *in vivo*. Our findings will provide the basis for new ideas regarding the prognosis and treatment of CC patients.

## Materials and Methods

### Data Processing and Screening of PCD-Related Genes

Transcriptome sequencing data and clinical information on CC patients were downloaded from The Cancer Genome Atlas (TCGA) (https://www.cancer.gov/tcga/). Patients with incomplete clinical pathological information and missing survival data were excluded. We finally followed up 436 CC patients. We downloaded RNA-seq TCGA transcriptome data in a standardized fragment format per kilobase exon model per million mapped reads. **Supplementary Table S1** shows the clinical information of all patients.

### Identification of PCD-Related lncRNAs

To obtain the lncRNA expression profiles, we mapped TCGA sequencing data to lncRNA annotation files in the GENCODE database (http://www.gencodegenes.org) and eliminated lncRNAs with no expression in > 20% of samples. We screened PCD-related lncRNAs in TCGA-Colon Adenocarcinoma (COAD) data using Spearman correlation analyses based on correlation coefficients > 0.3 and P < 0.05 (**Supplementary Table S2**). The top 10 lncRNAs for differential PCD genes were visualized using Cytoscape to explore the prognostic value of PCD-related lncRNAs, and those with prognostic value were screened using univariate Cox regression analysis. The expression of PCD-related lncRNAs in CC and normal tissues was analyzed using Wilcoxon signed-rank tests.

### Analysis of CC Subtypes Defined by PCD-Related lncRNAs

We used the ConsensusClusterPlus package in R to determine the number of consistent clusters based on the expression PCD-related prognostic lncRNAs and their potential biological characteristics. Prognostic value and clinicopathological characteristics in subgroups were analyzed using Kaplan–Meier (KM) survival analysis and log-rank tests.

### Biological Functions and Immune Infiltration Level Analysis Among Different Subgroups

Differential genes among subgroups were screened using the limma package in R with the following criteria: |logFC| ≥ 0.5 and a false discovery rate (FDR) < 0.05 (**Supplementary Table S3**). Differentially expressed genes (DEGs) among subgroups were analyzed using Gene Ontology (GO), the Kyoto Encyclopedia of Genes and Genomes (KEGG), clusterProfiler, org.Hs.eg.db, and the enrichplot package in R to determine their biological functions. Differences in tumor signal pathways between patients in clusters 1 and 2 were screened *via* Gene Set Enrichment Analysis (GSEA) using FDR < 0.05 as the criterion.

We calculated immune and stromal scores in CC to compare immune infiltration between the subgroups using the ESTIMATE algorithm. The abundance of immune cells was determined using TIMER (19), CIBERSORT (20, 21), QUANTISEQ (22), Microenvironment Cell Populations-counter (MCPCOUNTER) (23), XCELL (24), and the Estimating the Proportion of Immune and Cancer cells (EPIC) algorithm (25). Immune pathways in groups were quantified using the GSVA package in R. Differences in immune checkpoints between subgroups were evaluated using Wilcoxon tests.

### Construction of PCD-Related lncRNA Risk Model of CC

We constructed a prognostic model by screening eight PCD-related lncRNAs and then optimized readability using the LASSO regression algorithm based on their expression and corresponding risk coefficients. The formula was calculated as follows:


Risk score = ∑regression coefficient(gene i)   ×expression value(gene i)

The training and validation sets were divided into high- and low-risk groups based on the median risk score. The overall survival (OS) of patients was assessed using KM curves and log-rank tests. The effectiveness of the model was evaluated using receiver operator characteristic (ROC) curves. Whether risk scores and clinical characteristics were independent prognostic factors for patients were evaluated using univariate and multivariate Cox analyses. Differences in risk scores, immune scores, and clinicopathological characteristics were assessed using Wilcoxon tests. We used the RMS package in R to integrate clinical characteristics with risk scores and construct a nomogram and calibration curve to realize a quantitative prognostic tool. Consistency between the prognostic assessment by the nomogram and actual results was evaluated using a calculation with a guide method that included 1,000 resamples. The diagnostic power of the nomogram and individual predictors was evaluated using ROC curves. The clinical benefits conferred by prognostic evaluation of the nomogram and a single predictor were further compared using decision curve analysis (DCA).

### Analysis of TMB and Gene Copy Number Variation Among Subgroups

Somatic CC mutation data were retrieved from TCGA (https://www.cancer.gov/tcga/). The TMB was calculated by dividing the total number of mutations by the size of the coding region of the target gene. Patients were classified as having high or low TMB based on the median value. We also visualized the top 20 genes with the highest mutation frequency in high- and low-risk groups using the maftools package in R. Copy number variations (CNV) between subgroups were calculated using Chi-square tests, and the positions of CNVs on the chromosome were visualized using the Rciorcos package in R.

### Immunotherapy and Targeted Drug Screening

The sensitivity of subgroups of patients to immunotherapy was assessed using http://tide.dfci.harvard.edu. Based on the Genomics of Drug Sensitivity in Cancer (https://www.cancerrxgene.org), we used the pRRophetic package in R to evaluate the sensitivity of patients to chemotherapeutic agents and visualized the three-dimensional (3D) molecular structure of each agent using the cMAP database (https://clue.io/cmap).

### Source of Clinical Samples

We collected CC tumor and adjacent cancerous tissue from patients who underwent CC resection between 2016 and 2021 at the Colorectal Cancer Center of Shanghai Tenth People’s Hospital. After excluding patients with incomplete clinical data, 96 were included in the follow-up analysis. The study was approved by the Ethics Committee at Shanghai 10th People’s Hospital.

### Extraction of RNA and Real-Time Quantitative Polymerase Chain Reaction (RT-qPCR)

Total RNA from tissues or cells was extracted using TRIzol reagent (Invitrogen, Carlsbad, CA, USA) and quantified using a Nanodrop 2000 spectrophotometer (Thermo Fisher Scientific Inc., Waltham, MA, USA). Total RNA was reverse transcribed to cDNA using the PrimeScript™ RT Reagent Kit (Takara Bio Inc., Kusatsu, Japan). Amplicons were quantified using a 7500 Fast Real-time PCR system (Applied Biosystems, Piscataway, NJ, USA). The relative expression (fold change) of the target genes was determined using the 2-ΔΔCT method. Glyceraldehyde 3-phosphate dehydrogenase was the internal control. **Supplementary Table 4** shows the sequences of the RT-qPCR primers (Beijing Qingke Biotech Ltd., Beijing, China).

### Cell Culture and Transfection

Human HCT-116 and SW480 CC cell lines (American Type Culture Collection, Manassas, VA, USA) were cultured in Dulbecco’s modified Eagle’s medium containing 10% fetal bovine serum (FBS; both from Thermo Fisher Scientific Inc., Waltham, MA, USA) with 1% streptomycin and penicillin, at 37°C under a humidified 5% CO_2_ atmosphere.

Cells were transiently transfected using Lipofectamine 2000 (Invitrogen) as described by the manufacturer. Small interfering RNA (siRNA) was purchased from GenePharma (Shanghai, China) The sense and anti-sense si-U62317.4 sequences were: 5′-GAAGAGAAGGACAAGUUGACG-3′ and 5′-UCAACUUGUCCUUCUCUUCUG-3′, respectively. We created a stable knockout U62317.4 cell line using short hairpin RNA (shRNA) targeting U62317.4 (sh-U62317.4; General Biosystems, Anhui, China) with the sense and anti-sense sequences, 5′-GATACTTGATCCTGATAAA-3′ and 5′- TTTATCAGGATCAAGTATC-3′, respectively.

Lentiviral particles were obtained as described (16). HCT-116 cells were directly infected with Polybrene (Santa Cruz Biotechnology, Dallas, TX, USA) for 24 h and then, transfected cells were screened for 7–10 days with 2 μg/mL of puromycin (Invitrogen).

### Cell Proliferation and Colony Analysis

We transfected HCT-116 or SW480 cells for 48 h and then seeded 1 × 10^3^ cells/well in 96-well plates. After 24, 48, and 72 h, the cells were incubated with CCK-8 reagent (10 µL) in serum-free medium (100 µL) for 2 h at 37°C. The optical density at 450 nm was measured using a microplate reader. Similarly transfected CC cells were seeded in six-well plates and cultured in DMEM medium containing 10% FBS for 7 days to analyze colony formation. The cells were then fixed with paraformaldehyde and stained with 0.1% crystal violet. Colonies were counted using ImageJ software.

### Cell Invasion, Migration, and Wound Healing Assay

Cell invasion and migration were measured using Transwell chambers (Corning CoStar, Tewksbury, MA, USA). Cells (4 × 10^4^) were resuspended in serum-free medium and added to the upper part of Matrigel^®^-coated chambers. Medium (500 µL) containing 10% FBS was added to the lower chamber. The cells were fixed with paraformaldehyde 48 h later and stained with 0.5% crystal violet. Cells that passed through the bottom of the membrane were assessed and counted using a microscope. The cells were resuspended in serum-free medium and seeded in six-well plates until they reached 100% confluence, when they were damaged by being gently scratched with a 200-μL sterile micropipette tip for wound healing assays. Representative images of cell migration were acquired after 0, 24, and 48 h, and the cell migration rate was determined by time-lapse analysis using ImageJ software.

### Cell Apoptosis Assay

We assayed apoptosis using Flow Apoptosis Kits (BD Biosciences, San Diego, CA, USA) as described by the manufacturer. Briefly, digested cells were washed twice with cooled PBS and stained using FITC Annexin V apoptosis detection kits (BD Biosciences); thereafter, cell populations were evaluated using a BD LSRFortessa™ analyzer (BD Biosciences).

### Tumor Xenotransplantation

Four-week-old female BALB/C nude mice were randomly assigned to two groups (n = 5 per group) to determine tumor formation *in vivo*. CC cells stably transfected with sh-normal control (NC) or sh-U62317.4 were implanted subcutaneously into the axillae of nude mice. One week after injection, tumor volumes (cm^3^) were measured every 3 days as (length × width^2^)/2. The mice were euthanized 21 days later, and tumors were removed and weighed. The Animal Experiment Ethics Committee of Tongji University approved all the animal experiments.

### Western Blotting

Proteins were collected from CC cells in RIPA lysis buffer (Invitrogen) containing phenylmethylsulfonyl fluoride (PMSF; Bio-Rad Laboratories Inc., Hercules, CA, USA). Proteins were resolved by 10% sodium dodecyl sulfate-polyacrylamide gel electrophoresis and then transferred onto polyvinylidene difluoride membranes (Invitrogen). Non-specific antigen binding was blocked by shaking the membranes in 5% skim milk for 2 h at 37°C. Thereafter, the membranes were incubated with primary antibody overnight at 4°C, followed by secondary antibody for 2 h. The blots were visualized and analyzed using the Odyssey system (LI-COR Biosciences, Lincoln, NE, USA). **Supplementary Table 5** shows details of the antibodies.

### Statistical Analyses

Data were statistically analyzed, and images were generated using R and GraphPad Prism 8.03 (GraphPad Software Inc., San Diego, CA, USA). The Wilcoxon signed-rank and Kruskal-Wallis tests were used for between-group comparisons. Values with P < 0.05 were considered statistically significant.

## Results

### Consensus Clustering in CC Identified Prognostically Distinct Clusters Based on PCD-Related lncRNAs

**Figure 1:**
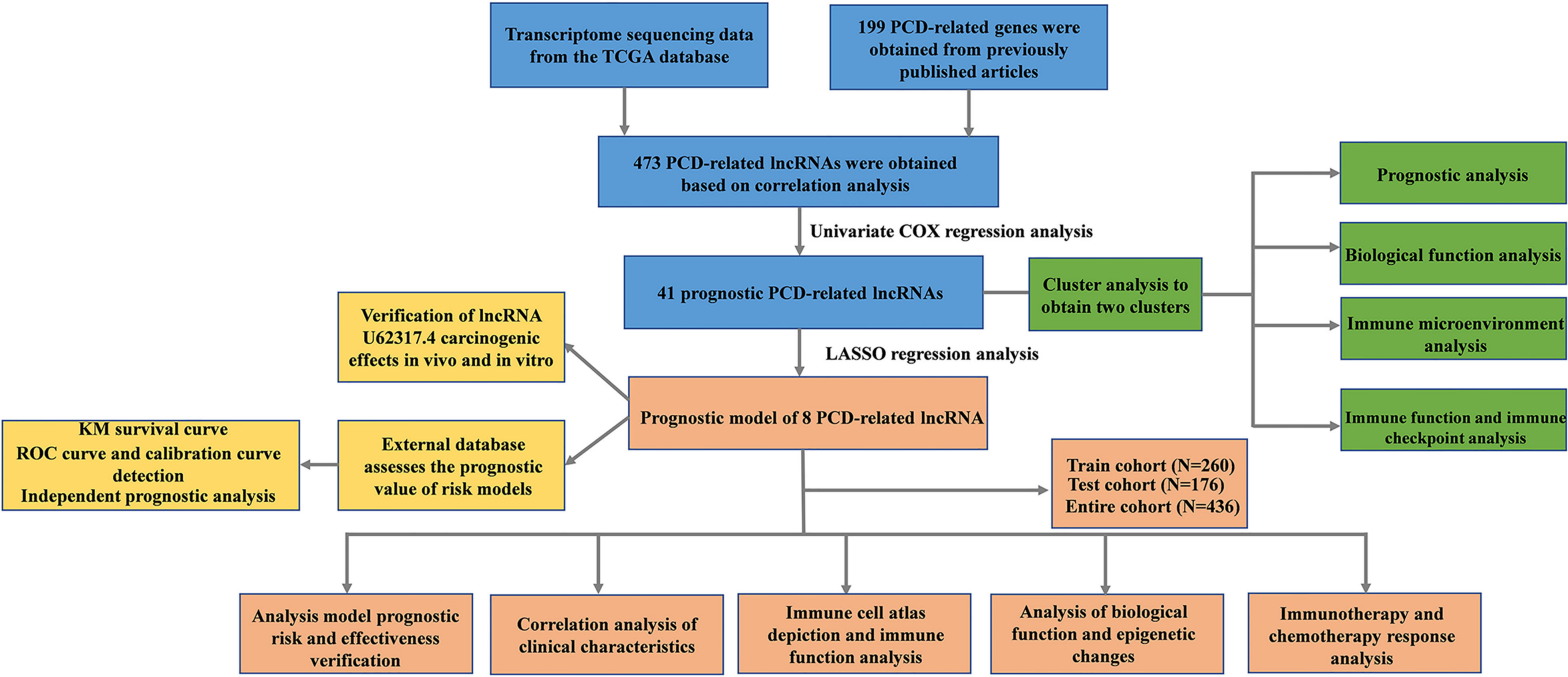
Flow chart of the study.

**Figure 2:**
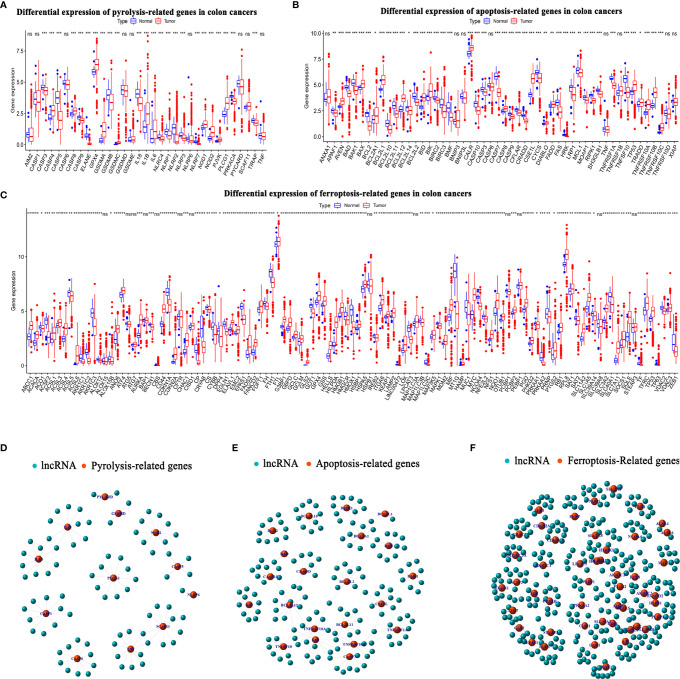
Long noncoding RNAs (lncRNAs) associated with PCD in colon cancer. **(A–C)** Expression levels of PCD-related genes in TCGA-COAD cancer database in CC and normal groups. **(A)** Pyroptosis- **(B)** apoptosis-, and **(C)** ferroptosis-related genes. **(D–F)** Top 10 lncRNAs in TCGA-COAD database associated with differentially expressed PCD genes. **(D)** Pyroptosis-, **(E)** apoptosis-, and **(F)** ferroptosis-related lncRNAs. CC, colon cancer; COAD, colon adenocarcinoma; PCD, programmed cell death; TCGA, The Cancer Genome Atlas. *P < 0.05, **P < 0.01, ***P < 0.001, ns, non-significant.

**Figure 3:**
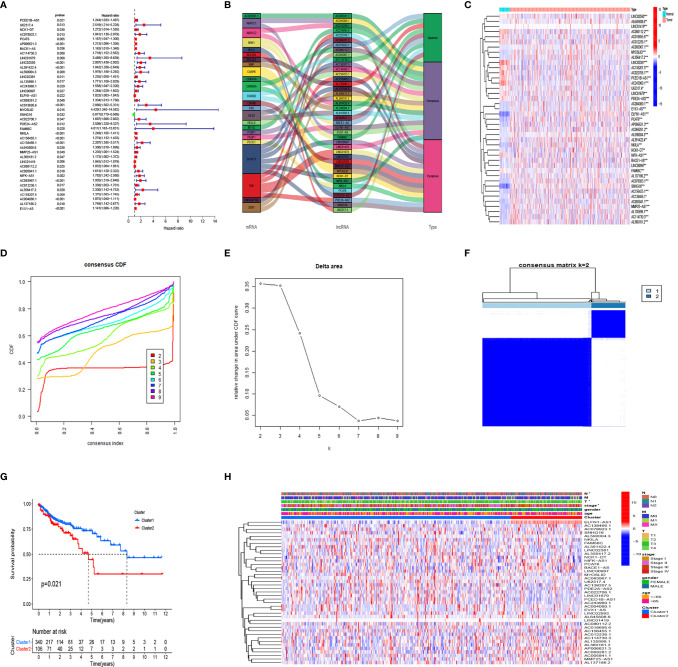
Consensus cluster analysis of PCD-related lncRNA in colon cancer. **(A)** Prognostic value of 41 PCD-related lncRNAs. **(B)** Relationships among prognostic-related lncRNA, mRNA, and type of death. **(C)** Expression of PCD prognostic-related lncRNA in CC and normal tissues. **(D–F)** Cumulative distribution function. **(D)** change in area under the CDF curve, **(E)** for k=2-9, **(F)** Consensus clustering matrix for k = 2. **(G)** Kaplan–Meier survival analysis of patients in clusters 1 and 2. **(H)** Clinicopathological characteristics and lncRNA expression in clusters 1 and 2. CC, colon cancer, CDF, cumulative distribution function; PCD, programmed cell death. *P < 0.05, **P < 0.01, ***P < 0.001.

**Figure 1** shows a flow chart of the study. We initially re-annotated TCGA expression matrix to distinguish between mRNAs and lncRNAs and identified 199 genes related to PCD among which, 33, 52, and 114 were associated with apoptosis (26), pyroptosis (9, 27), and ferroptosis (28). **Figures 2A–C** shows that the expressions of most PCD-related genes significantly differed between CC and normal tissues. We then screened the lncRNAs that were the most closely associated with the expression of these genes using correlation analysis. Among the 437 PCD-related lncRNAs, 85, 161, and 297 were associated with pyroptosis, apoptosis, and ferroptosis, respectively (**Figures 2D–F**). Univariate Cox analysis of the screened PCD-lncRNAs identified 41 PCD prognosis–related lncRNAs (**Figures 3A, B**). Differential analysis showed that the expression of these lncRNAs significantly differed between tumor and normal tissues (**Figure 3C**). Consensus clustering based on the expression of these lncRNAs revealed biological differences among different CC subgroups. Combining the consensus matrix cumulative distribution function curve and the delta area plot showed that interference between clusters was minimal and the classification was significant when K = 2 (**Figures 3D–F**). KM survival curves showed that the OS rate was significantly better for cluster 1 than that cluster 2 (**Figure 3G**). We then constructed a heat map to compare the clinical characteristics between these subgroups and found high scores for T stage, lymphatic metastasis, and tumor stage in cluster 1 (**Figure 3H**).

### Analysis of Biological Functions Between CC Subgroups

**Figure 4:**
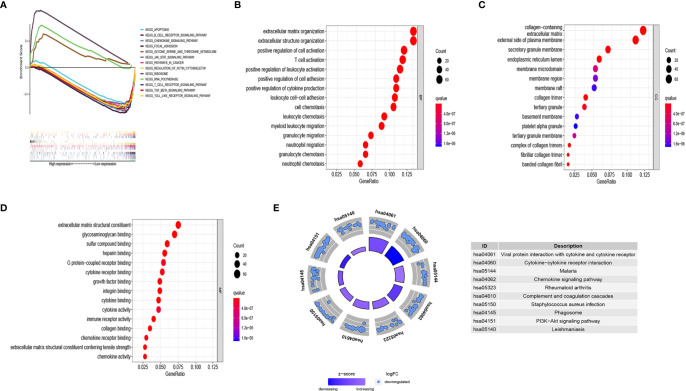
Analysis of the biological function of differentially expressed genes (DEGs) in different clusters. **(A)** KEGG enrichment of cluster 1 and cluster 2 by GSEA. Only gene sets with FDR q < 0.05 were considered significant. **(B)** Biological process (BP) analysis of DEGs. **(C)** Cellular component (CC) analysis of DEGs. **(D)** Molecular function (MF) analysis of DEGs. **(E)** KEGG pathway enrichment analysis of DEGs. Only gene sets with FDR q < 0.05 were considered significant.

The survival results revealed evident prognostic differences between the CC subgroups. To explore the potential reason for this, we analyzed DEGs in the subgroups using GSEA, GO, and KEGG. The GSEA results showed that the immune-related B cell receptor, chemokine, JAK-STAT, T cell receptor, and Toll-like receptor signaling pathways were abnormally activated in cluster 1 (**Figure 4A**). The GO results showed that the DEGs were involved in several immune-related functions, which was consistent with the GSEA findings (**Figures 4B–D**). The KEGG analysis showed that the DEGs were primarily involved in cell growth, chemokines, cytokines, and phagosomes related to signaling pathways (**Figure 4E**).

### Analysis of the Tumor Immune Microenvironment and Immune Checkpoint Genes Clusters 1 and 2

**Figure 5:**
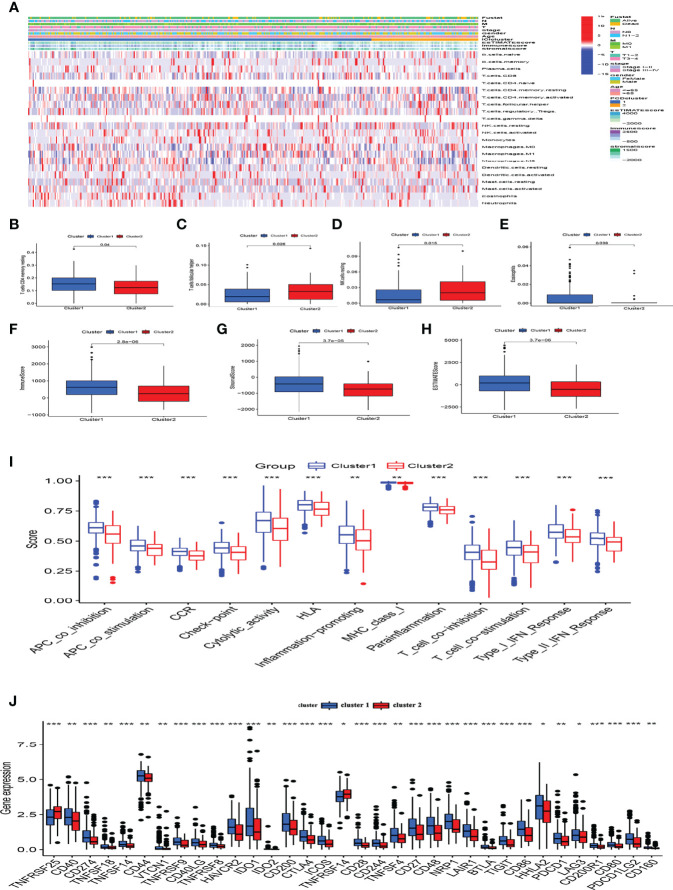
Analysis of the immune microenvironment and immune functions in different clusters. **(A)** Panoramic view of immune microenvironment in different clusters. **(B–E)** CIBERSORT analysis of differences in immune cells among clusters, only P < 0.05 was considered statistically significant. **(F–H)** Immune, stromal and ESTIMATE scores among subgroups. **(I)** Immune function scores in different clusters. **(J)** Expression of immune checkpoints in clusters. *P < 0.05, **P < 0.01, ***P < 0.001.

Considering that the DEGs were primarily related to immune-related functions, we examined the immune cell distribution in the immune microenvironment of CC. The amounts of immune cell infiltration differed between clusters 1 and 2 (**Figure 5A**). Cluster 1 had abundant memory CD4 T cells and eosinophils, whereas cluster 2 had more follicular helper T and resting natural killer (NK) cells (**Figures 5B–E**). We further explored differences in the distribution of immune and stromal ratios between the two clusters using the ESTIMATE algorithm. **Figures 5F–H** shows that cluster 2 had lower immune, stromal, and ESTIMATE scores than those of cluster 1. A comparative analysis revealed fully suppressed immune function in cluster 2 (**Figure 5I**). Considering the importance of immune checkpoints in immunotherapy, we analyzed the expression of immune checkpoints in the two clusters and found that all immune checkpoint genes except the TNF Receptor Superfamily Members *(TNFRSF) 25* and *14* were significantly suppressed in cluster 2 (**Figure 5J**). Thus, the lower OS rate of patients in this cluster might have been due to suppressed immune function.

### Construction of a PCD-Related lncRNA Prognostic Model for CC Patients Using TCGA

**Figure 6:**
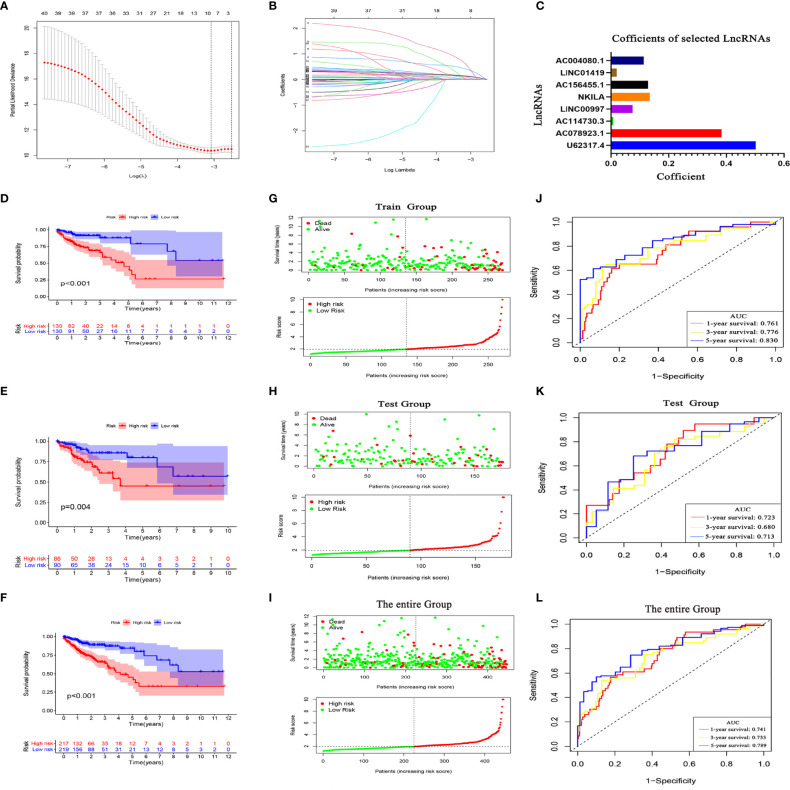
Programmed cell death (PCD)-related lncRNA risk prognostic model. **(A)** Coefficient spectrum of PCD-related lncRNAs. **(B)** Trajectory of correlation coefficients of factors increasing with Log λ. **(C)** Coefficients of eight PCD-related lncRNAs. Kaplan–Meier survival curves in TCGA training **(D)**, test **(E)**, and entire **(F)** sets. Distribution of risk scores and survival status in training **(G)**, test **(H)**, and entire **(I)** sets. evaluation of prognostic model effectiveness in training **(J)**, test **(K)**, and entire **(L)** sets.

LASSO Cox analysis of PCD prognosis–related lncRNAs in TCGA identified the filter genes AC004080.1, AC078923.1, AC114730.3, AC156455.1, long intergenic non-protein coding RNA 1419 (LINC01419), LINC00997, NF-kappaB interacting lncRNA (NKILA), and U62317.4 with which to construct a prognostic model. Risk scores were calculated based on the regression coefficients and expression of these eight genes (**Figures 6A–C**). The risk formula was 0.5023 * expression (U62317.4) + 0.3836 * expression (AC078923.1) + 0.0082 * expression (AC114730.3) + 0.075 * expression (LINC00997) + 0.1341 * expression (NKILA) + 0.1278 * expression (AC156455.1) + 0.0195 * expression (LINC01419) + 0.1131 * expression (AC004080.1). To improve the accuracy and effectiveness of the prognostic model, we randomly assigned the patients to training (N = 260) and test (N = 176) groups and then divided them into high-and low-risk groups according to the median risk score. The KM survival curves indicated worse OS for patients in the training group with high risk scores than those with low risk scores (**Figure 6D**). The risk curves indicated a significantly higher death rate among patients with high risk scores than those with low risk scores (**Figure 6G**). The ROC curves showed that the prognostic model accurately predicted the 1, 3, and 5-year survival rates of CC patients (**Figure 6J**). This model was further verified in the test group (**Figures 6E, H, K**) and the entire TCGA database (**Figures 6F, I, L**). These findings indicated that our prognostic model is unbiased and can be used as a reference tool for predicting the OS rate of CC.

### Analysis of Independent Factors and Clinicopathological Correlations of the Prognostic Model

**Figure 7:**
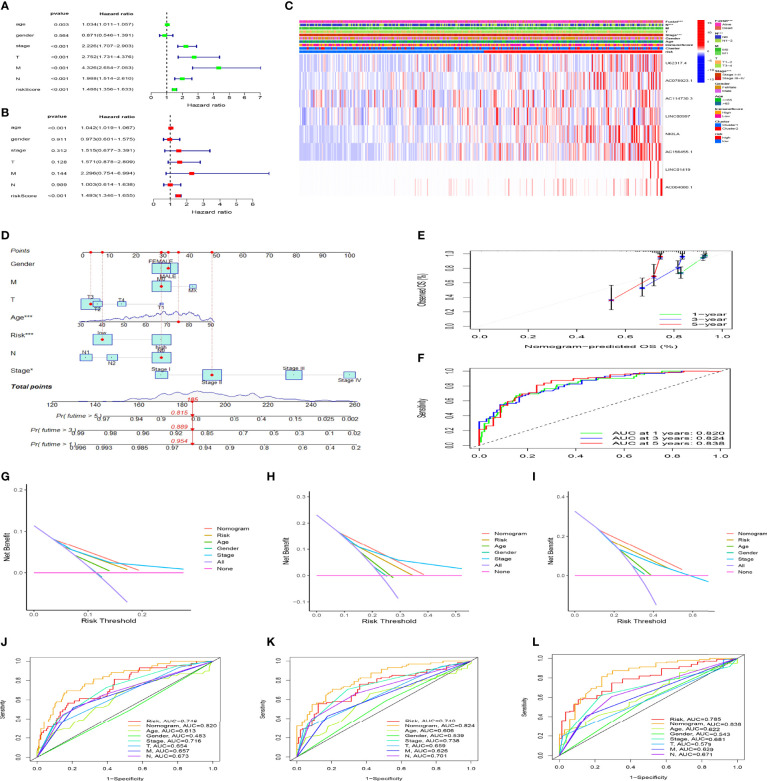
The risk model constructed using programmed cell death (PCD)-related lncRNAs is an independent prognostic factor for colon cancer. Cox univariate **(A)** and multivariate **(B)** analyses of clinical characters and risk scores. **(C)** Heat maps of correlations among clinical characteristics, immune score clusters, and risk scores. **(D)** Nomogram for clinical prognosis assessment. **(E)** Calibration curve to evaluate nomogram accuracy. **(F)** Assessment of prognostic effect of nomogram. **(G-I)** Evaluation of clinical benefits of nomogram. **(J-L)** Comparison of prognostic efficacy of single prognostic factor and nomogram. *P < 0.05, ***P < 0.001.

We further confirmed the independence of the CC prognostic model using univariate and multivariate Cox analyses. **Figures 7A, B** shows that age (hazard ratio [HR], 1.042; 95% confidence interval [CI], 1.019–1.067; P < 0.001) and risk score (HR, 1.493; 95% CI, 1.346–1.655; P < 0.001) were independent prognostic factors for CC patients. We then evaluated the relationship between the risk score and PCD prognosis–related lncRNA. A heatmap showed significantly increased expression of all eight PCD-related lncRNAs in the high-risk group (**Figure 7C**). Risk scores were statistically different in cluster stratification, tumor stage, and lymph node metastasis (**Supplementary Figure 1A**). In addition, risk scores could assess the prognosis of patients in multiple clinical subgroups, except for patients in the T1-2 group (**Supplementary 1B**). We constructed a nomogram that included patient age, sex, tumor stage, distant metastasis, lymph node metastasis, and risk score to accurately quantify survival rates (**Figure 7D**). The calibration curves showed that the actual 1-, 3-, and 5-year OS rates of patients and those estimated by the nomogram were close (**Figure 7E**). The areas under the ROC curve (AUC; **Figure 7F**) revealed that the 1, 3, and 5-year survival rates determined by the nomogram were accurate (AUC = 0.820 0.824, and 0.838, respectively). The DCA decision curve showed that the net rate of return for the OS rates assessed by the combined model was better than other clinical characteristics (**Figures 7G–I**). The ROC curve also showed that the combined model was more sensitive than other clinical features (**Figures 7J–L**). These results showed that our nomogram can help clinicians plan accurate follow-up strategies.

### Analysis of Functional Enrichment and Immune Cell Infiltration Levels in High- and Low-Risk Groups

We explored the potential biological functions of and signaling pathways enriched by the DEGs in the high- and low-risk groups (**Supplementry Table 6)**. The GO results showed that DEGs were significantly enriched in the processes of epithelial-mesenchymal transition, Wnt signaling pathway regulation, and cytokine stimulation (**Supplementary Figure 2A**). The KEGG results revealed that DEGs were primarily involved in Wnt, Hippo, extracellular matrix-receptor interactions, transforming growth factor-beta, and cytokine-cytokine receptor interaction signaling pathways (**Supplementary Figure 2B**).

**Figure 8:**
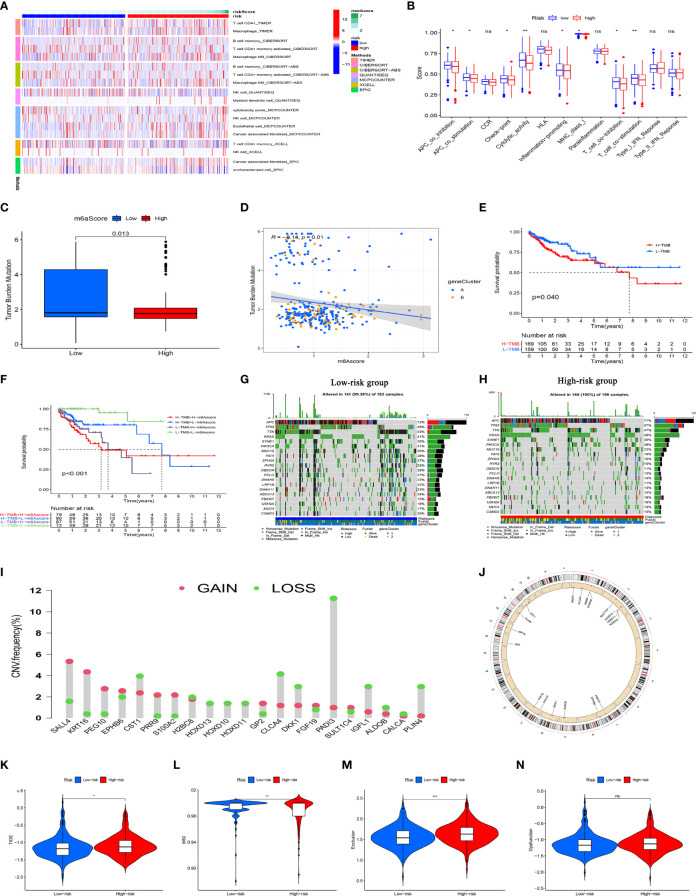
Immune response, TMB, CNV, and immunotherapy analysis of high- and low-risk groups. **(A)** Immune cell infiltration analysis among risk groups using TIMER, CIBERSORT, QUANTISEQ, MCPCOUNTER, XCELL, and EPIC algorithms. All immune cells shown statistically differed. **(B)** Immune function scores for high- and low-risk groups. **(C)** Tumor metastasis burden differs between high- and low-risk groups. **(D)** Scatter plot of negative correlation between risk score and TMB. **(E)** Survival analysis of high-and low TMB in TCGA database. **(F)** Survival analysis of TCGA colon cancer stratified by TMB and risk score. **(G, H)** Waterfall chart shows more genes with increased mutation frequency in high- and low-risk groups. **(I)** Distribution of CNV frequency among DEGs in the high- and low-risk groups. Green and red, deletion and amplification, respectively. **(J)** Distribution of DEGs with different CNVs on chromosomes. **(K–N)** TIDE, MSI, and T cell exclusion and dysfunction scores in high- and low- risk group., *P<0.05, **P<0.01, ***P<0.001; ns, not significant. CNV, copy number variations; DEGs, differentially expressed genes; MSI, microsatellite instability; TCGA, The Cancer Genome Atlas; TIDE, tumor immune dysfunction and exclusion; TMB, tumor mutation burden.

**Figure 8A** shows an immune infiltration heat map based on the TIMNER, CIBERSORT, QUANTISEQ, MCPCOUNTER, XCELL, and EPIC algorithms. Among immune functions, difference analysis showed that Apcin (APC) co-inhibition, APC co-stimulation, checkpoint, cytolytic activity, inflammation promotion, MHC class I, T cell co-inhibition, and T cell co-stimulation significantly differed between the high-and low-risk groups (**Figure 8B**). These findings showed that the eight PCD-related lncRNA prognostic characteristics of CC are somewhat related to immune cell infiltration.

### Correlation of Risk Score With TMB and Gene CNVs

In view of the evident prognostic differences in the high- and low-risk patients, we analyzed the TMBs and CNVs to further uncover underlying causes. **Figure 8C** shows lower TMB levels in patients with high risk scores than those with low risk scores. Further correlation analysis showed that the risk score negatively correlated with TMB (**Figure 8D**). We then divided the patients into groups with a high or low TMB based on the median TMB. KM curves revealed a significantly worse OS rate for patients with a high TMB than those with a low TMB (**Figure 8E**). Considering that the risk score and the TMB have good prognostic value in CC patients, we evaluated the synergistic effects of these scores on the prognostic stratification of CC. We found that the TMB did not affect the assessment of the prognosis of CC patients according to risk score. Survival in risk score subtypes significantly differed between groups with a high or low TMB (**Figure 8F**). These results indicated that the risk score might be an effective indicator that can evaluate prognosis independently of the TMB. We assessed the distribution of somatic variations in CC driver genes between the high- and low-risk groups. A waterfall chart shows the top 20 genes with the highest mutation frequency (**Figures 8G, H**). Our analysis of mutation annotation files in TCGA cohort revealed that FAT4, OBSCN, PCLO, ABCA13, ZFHX4, DNAH11, RYR2, and USH2A significantly differed between the high- and low-risk groups (**Supplementary Table 7**). In addition, we performed CNV analysis of the top 40 genes with the greatest differences between the high- and low-risk groups. We found a higher frequency of CNV mutations in DEGs between the high-and low-risk groups, where CNV expansion occurred in SALL4, KRT16, PEG10, EPHB6, PRR9, GP2, FGF19, and SULT1C4, whereas CST1, H2BC8, HOXD10, HOXD10, HOXD11, CLCA4, DKK1, IGFL1, ALDOB, CALCA, and PLIN4 underwent CNV deletion (**Figure 8I**). The locations of CNV mutations in these DEGs are shown in **Figure 8J**. These results may provide new ideas for studying gene mutations in PCD-related lncRNAs in CC.

### Benefits of Immunotherapy to High- and Low-Risk Groups

We evaluated the efficacy of immunotherapy in the high- and low-risk groups using the website http://tide.dfci.harvard.edu/. A higher tumor immune dysfunction and exclusion (TIDE) score implies a higher possibility of immune escape, indicating that the patient has a lower benefit from immunotherapy. As shown in **Figure 8K** and **Supplememtary Table 8**, the TIDE scores of patients in the high-risk group were significantly higher than those in the low-risk group, implying that the low-risk group patients would benefit from immunotherapy. Patients in the high-risk group had lower microsatellite instability (MSI) scores (**Figure 8L**), whereas patients in the low-risk group had higher T cell exclusion scores (**Figure 8M**). T cell dysfunction did not significantly differ between the subgroups (**Figure 8N**). These results support a basis for novel customized immunotherapies for CC patients.

### Analysis of Drug Sensitivity Potential in High- and Low-Risk Groups

**Figure 9:**
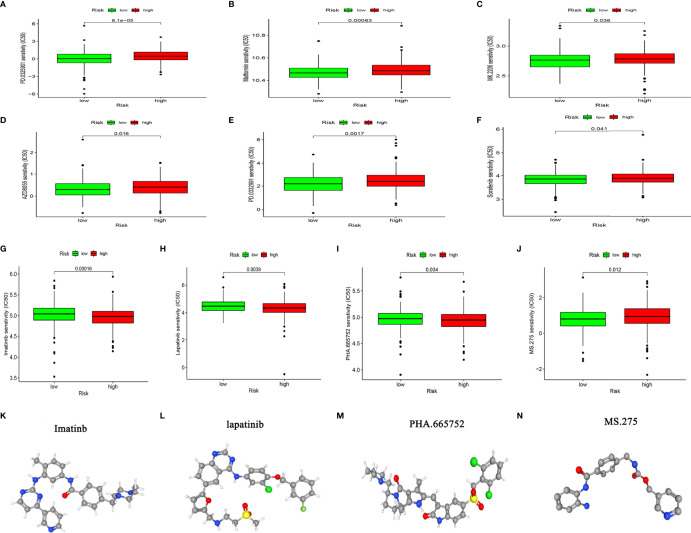
Chemosensitivity analysis. **(A–J)** Responses of high- and low-risk groups to chemotherapy drugs. **(K–N)** Three-dimensional structure of small molecule drugs.

Considering that chemotherapy and targeted therapies are popular strategies for treating CC, understanding the sensitivity of subgroups of patients to such drugs is important. We predicted the sensitivity of the high-and low-risk groups to agents that are commonly administered to CC patients. **Figures 9A–F** shows that the group with low-risk scores was more sensitive to PD.0325901 METFORMIN MK.2206 AZD8055 PD.0332991, and sorafenib, whereas that with high-risk scores was more sensitive to imatinib, lapatinib, PHA.665752, and MS.275 (**Figures 9G–J**). We determined the 3D structure of four chemotherapy drugs that could be used for patients in the high-risk group using the CMAP database (**Figures 9K-N**). These results should facilitate the application of precise and or new personalized medicines for treating CC.

### External Database Verified the Prognostic Value of the Risk Model Constructed With PCD-Related lncRNAs

**Figure 10:**
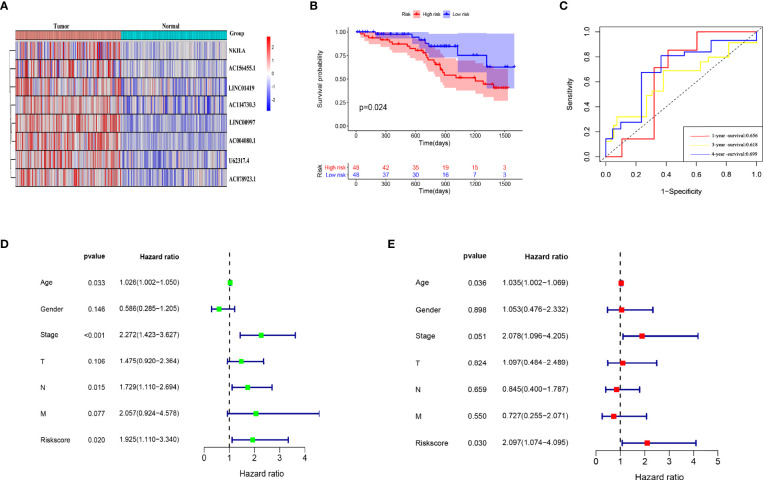
External database verification that risk model has prognostic value. **(A)** Model lncRNA expression in 96 CC and adjacent tissues. **(B)** Kaplan-Meier Survival analysis of patients in high- and low-risk groups. **(C)** Evaluation of risk model effectiveness. **(D, E)** Univariate and multivariate regression analyses of clinical characteristics and risk scores.

We selected 96 CC patients from the Colorectal Cancer Center of Shanghai Tenth People’s Hospital to verify the external dataset and examined the expression of the eight PCD-associated lncRNAs in CC and adjacent tissues by RT-qPCR. The expression trends were consistent with those listed in TCGA (**Figure 10A**). We calculated the risk scores of the patients and divided them into high- and low-risk groups according to the median risk score. KM curves showed that the OS was significantly worse in the high- risk group than that in the low-risk group (**Figure 10B**). The AUC showed that the risk model could predict the outcome of CC patients (**Figure 10C**). Univariate and multivariate Cox regression analyses of clinical characteristics and risk scores determined that risk scores, stage and age were independent prognostic factors (**Figures 10D, E**).

### Silencing U62317.4 Inhibits the Proliferation, Invasion, and Migration of CC

**Figure 11:**
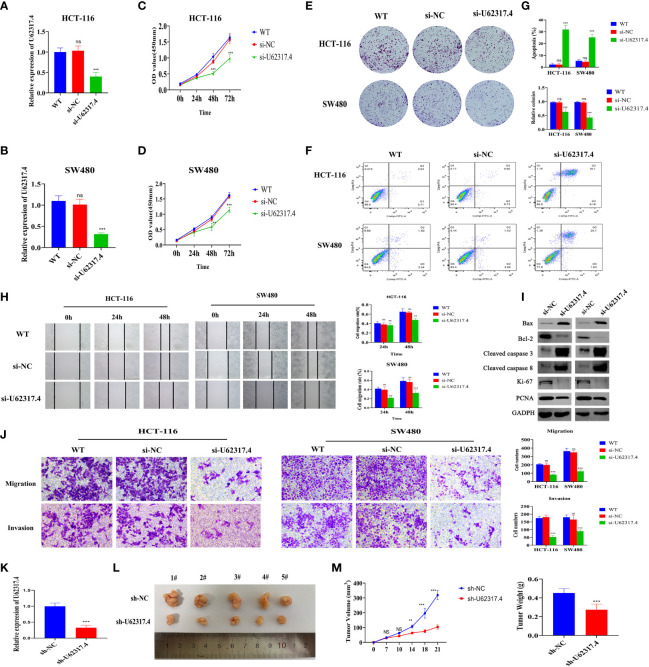
Silencing U62317.4 suppressed the malignant CC phenotype. **(A, B)** Detection of U62317.4 expression in HCT-116 **(A)** and SW480 **(B)** cells using RT-qPCR. **(C, D)** CCK-8 analysis of HCT-116 **(C)** and SW480 **(D)** cells transfected with si-NC or si-U62317.4, and wild-type cells. **(E)** Colony formation of HCT-116 and SW480 cells transfected with si-NC, si-U62317.4, and wild-type cells. **(F)** Flow cytometry of HCT-116 and SW480 transfected si-NC or si-U62317.4, and wild-type cells. **(G)** Numbers of cell clones and apoptosis rates from three independent experiments. **(H)** Evaluation of migration abilities of HCT-116– and SW480-transfected si-NC or si-U62317.4, and wild-type cells using wound scratch assays. **(I)** Western blots of cell apoptosis and growth-related protein indicators. **(J)** Transwell assays of si-NC– or si-U62317.4–transfected HCT-116 and SW480 cell migration and invasion ability and wild-type cells **(K)** Efficiency of shRNA interference of U62317.4 confirmed *via* RT-qPCR. **(L, M)** Silencing U62317.4 significantly inhibits tumor weight and volume; n = 3. CC, colon cancer; NC, normal control. **P < 0.01, ***P < 0.001, ns, non-significant.

The findings that the lncRNA U62317.4 had the highest risk coefficient in the prognostic model indicated that U62317.4 is closely associated with the prognosis of CC. However, its role in the occurrence and development of CC is unclear. Therefore, we explored whether U62317.4 is involved in the malignant progression of CC. We synthesized a siRNA against U62317.4 and evaluated its interference *via* RT-qPCR. The expression of U62317.4 in HCT-116 and SW480 cells was significantly reduced after transfection with si-U62317.4 (**Figures 11A, B**). Silencing U62317.4 significantly inhibited CC cell viability and clone formation (**Figures 11C–E**) and significantly increased the apoptosis of CC cells (**Figures 11F, G**). Metastasis is another important feature of malignant tumors. Thus, we explored the role of U62317.4 in CC metastasis using wound healing and Transwell assays. Knockdown of U62317.4 significantly reduced wound healing ability as well as the migratory and invasive capacity of CC cells (**Figures 11H, J**). We constructed a nude mouse subcutaneous tumor model. to evaluate the tumorigenicity of U62317.4 *in vivo*. Silencing U62317.4 significantly inhibited the growth of CC in these mice (**Figures 11K–M**). Western blotting revealed changes in the abundance of proliferation- and apoptosis-related proteins when U62317.4 was silenced. **Figures 11I** shows that the expression of ki-67, PCNA, and BCL-2 decreased, whereas that of cleaved caspases-3 (cleaved casp-3) and -8 increased when U62317.4 was silenced in CC. These results showed that silencing U62317.4 inhibits CC cell proliferation and metastasis.

## Discussion

Apoptosis is a classical form of PCD that is considered the most promising target for tumor therapy. Pyroptosis and ferroptosis are important types of PCD-related death that are distinct from apoptosis. Their dysfunction is critical in the development of various tumors (6, 7, 29). Biomarkers of PCD play crucial roles in tumor prognosis assessment and tumor efficacy monitoring (30, 31). However, most previous studies have focused on genes that can be programmed for proteins. Considering the key roles of lncRNAs in PCD, their roles in the clinical features and prognosis of CC should be comprehensively evaluated.

We screened TCGA data and identified 41 lncRNAs associated with PCD in CC based on their prognostic value. We classified them into two subtypes based on their expression profiles. Prognostic evaluation and clinical correlation analysis showed that OS rate, tumor stage and tumor lymphatic metastasis significantly differed between these subtypes. We constructed a prognostic model comprising eight-PCD-related lncRNAs using LASSO regression analysis. We found that patients with high-risk scores had a poorer prognosis and that the prognostic model had high diagnostic power. In view of its importance in the prognostic model, we verified the roles of U62317.4 lncRNA *in vivo* and *in vitro*. Silencing U62317.4 lncRNA inhibited tumor proliferation and invasion, and promoted tumor apoptosis, indicating that the abundant expression of this gene is closely related to the progression of CC. We judged the prognostic significance of risk scores and clinical characteristics in CC using univariate and multivariate Cox regression analyses. Age and risk score were independent prognostic factors for CC patients. The results of the external database analysis provided further evidence that our model could accurately assess the prognosis of CC. The nomogram is a quantitative tool that can predict a certain clinical outcome or the probability of a certain type of event based on the values of several variables (32). The DCA decision and ROC curves showed a better net profit rate and diagnostic ability for the nomogram than those for a single indicator in terms of assessing patient prognosis. These findings indicated that this quantitative tool is important for patient disease management.

Cell death is an integral part of the immune response and serves as a signal (second messenger) to guide the immune system and the TME to ensure tissue repair and homeostasis (10, 33). A disordered immune system is an important cause of cancer progression, treatment failure, and eventual death (34). The significantly reduced immune microenvironment and immune function scores in cluster 2, implied suppressed immune function, which could explain the lower OS rate in this cluster. Many immune checkpoints (negative regulatory receptors) are expressed on T cells only after activation (35). Immune checkpoints influence the progression and treatment of CC (34, 36, 37). We found significantly reduced immune checkpoints in cluster 2, which might have been associated with the overall suppression of immune function. In addition, significantly more macrophage M0 and cancer-associated fibroblast infiltration was found in the high-risk group, which had significantly less NK and memory CD4 T cell infiltration that might have further exacerbated the immune depletion status of patients. Liu et al. found that NK cells can inhibit the proliferation of breast cancer cells by secreting perforin/granzyme to activate the apoptotic pathway (38). Nagorsen et al. (39) and Cui et al. (40) showed that increased M0 macrophage infiltration can inhibit the tumor immune activity of the digestive system and suggested that this leads to a poor prognosis. Cancer-related fibroblasts can tame immune cells to create a microenvironment suitable for tumor survival (41, 42). The loss of memory T lymphocytes further aggravates the exhaustion of T cells, which is also an important cause of immune dysfunction in patients with tumors (34, 43). These collective findings indicated that PCD-related lncRNAs impact the dysfunction of immune cells in CC immunity, thus providing a new platform for the development of novel immunotherapies.

A comprehensive assessment of PCD-related lncRNAs would help to understand the characteristics of immune cell infiltration and predict responses to immunotherapy. The TMB is a promising indicator of responses to immune checkpoint inhibitors, that closely correlate with immunity (44). We found that the TMB and MSI scores associated with sensitivity to immunotherapy were significantly lower in the high- risk group than those in the low-risk group. This was also consistent with the immunotherapy and immune status of the two subgroups assessed by the http://tide.dfci.harvard.edu website. The results of our stratified analysis showed that the risk model constructed using PCD-associated lncRNA was not associated with the TMB in CC. This means that the PCD-related risk model and TMB represent different aspects of tumor immunobiology and that the model can predict responses to immunotherapy independently of TMB.

Chemotherapy and immunotherapy are the most important adjuvant therapies for CC, as they can improve the prognosis and quality of life of patients. Considering the low immunogenicity and immunosuppression of patients in the high-risk group, we screened a batch of small molecule chemotherapeutics using the GDSC drug susceptibility database, with the aim of improving personalized medication guidance for CC patients. Based on the IC50 prediction, the high-risk group of patients was more sensitive to imatinib, lapatinib, PHA.665752, and MS.275. Dolloff et al. indicated that lapatinib can upregulate TRAIL receptors to induce CC cell apoptosis through off-target effects activated by the c-Jun N-terminal kinase and c-Jun pathways (45). The synthetic small molecule benzamide derivative of histone deacetylase inhibitor, MS-275 is currently in phase I/II clinical trials. This agent has demonstrated significant CC inhibition *in vitro* and *in vivo* (46, 47). The antitumor activity of MS-275 is primarily reflected in the induction of endogenous and exogenous apoptotic cell death in tumor cells (48). These results improve the guidance for personalized drug use in CC.

We provided a comprehensive view of the management of CC and we established a robust prognostic model. However, the present study has some limitations. TCGA was the main source of multi-omics data and clinical information. The results of the multi-omics analysis could not be verified. For example, we covered transcriptome sequencing, TMB, and CNV analyses. However, these tests are expensive and difficult to implement in practical applications. We hope that rapid advances in biotechnologies will lead to the development of robust toolkits that will pay the way for their widespread implementation. The TME might differ among tumor regions. However, most of the tissues that we analyzed were collected from core areas of tumors, which might have impacted our evaluation of TME characteristics and the immune functions of different tumor areas to some extent. We did not have any external data to validate the drug sensitivity results, which would undoubtedly likely be lengthy and expensive.

The present findings require further prospective validation by a multicenter study. Our study has some limitations. Nonetheless, we provided clues for elucidating the relationships between PCD-related lncRNAs and the TME as well as treatment responses. Our prognostic model has good clinical value and might lead to new ideas for improving the OS of CC patients and facilitate individualized treatment.

## Data Availability Statement

The original contributions presented in the study are included in the article/**Supplementary Material**. Further inquiries can be directed to the corresponding authors.

## Ethics Statement

The studies involving human participants were reviewed and approved by Shanghai Tenth People’s Hospital Ethics Committee. The patients/participants provided their written informed consent to participate in this study. The animal study was reviewed and approved by Animal Experiment Ethics Committee of Tongji University.

## Author Contributions

YC and ZC designed the project and analyzed the data. YZ and JL verified the results of the functional experiments. MZ, ML, WX, and ZL collected clinical samples and information. SZ and FS provided financial support. QW and ZC drafted and revised the manuscript. All authors agree with the publication of this manuscript.

## Funding

This work was supported by the Research Fund of Shanghai Municipal Health Bureau (No. 2019cxjq03), National Natural Science Foundation of China (Nos. 81930066, 81772941, and 82102449), Shanghai Tenth People’s Hospital Climbing Training Program (2021SYPDRC060), and Shanghai Municipal Health Commission (No. 20204Y0088).

## Conflict of Interest

The authors declare that the research was conducted in the absence of any commercial or financial relationships that could be construed as a potential conflict of interest.

## Publisher’s Note

All claims expressed in this article are solely those of the authors and do not necessarily represent those of their affiliated organizations, or those of the publisher, the editors and the reviewers. Any product that may be evaluated in this article, or claim that may be made by its manufacturer, is not guaranteed or endorsed by the publisher.

## Supplementary Material

Supplementary Figure 1 Significance of risk scores in clinical subgroups. **(A)** Distribution of risk scores and **(B)** prognostic evaluation of risk scores in clinical subgroups.

Supplementary Figure 2 Biological functions of differentially expressed genes (DEGs) in high- and low-risk groups. **(A)** Gene Ontology and **(B)** Kyoto Encyclopedia of Genes and Genomes analyses of DEGs in high- and low-risk groups.
