# Supplementary figures and images for: Characteristics of Prognostic Programmed Cell Death–Related Long Noncoding RNAs Associated With Immune Infiltration and Therapeutic Responses to Colon Cancer
Source: Front Immunol. 2022 May 31;13:828243. doi: 10.3389/fimmu.2022.828243 (PMC9195301; doi:10.3389/fimmu.2022.828243)

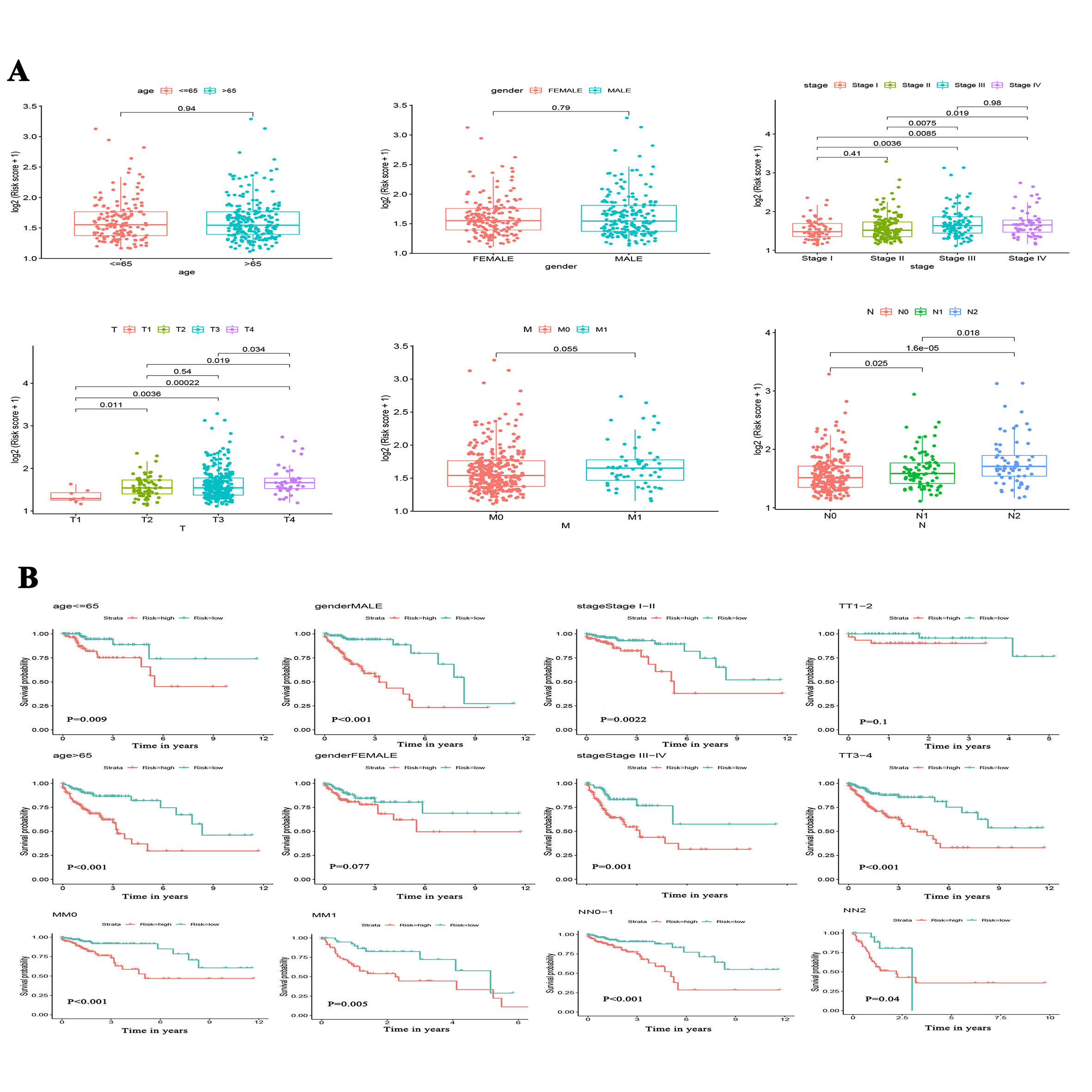

Supplement: Supplementary Figure 1 — Significance of risk scores in clinical subgroups. (A) Distribution of risk scores and (B) prognostic evaluation of risk scores in clinical subgroups. [file Image_1.jpeg]

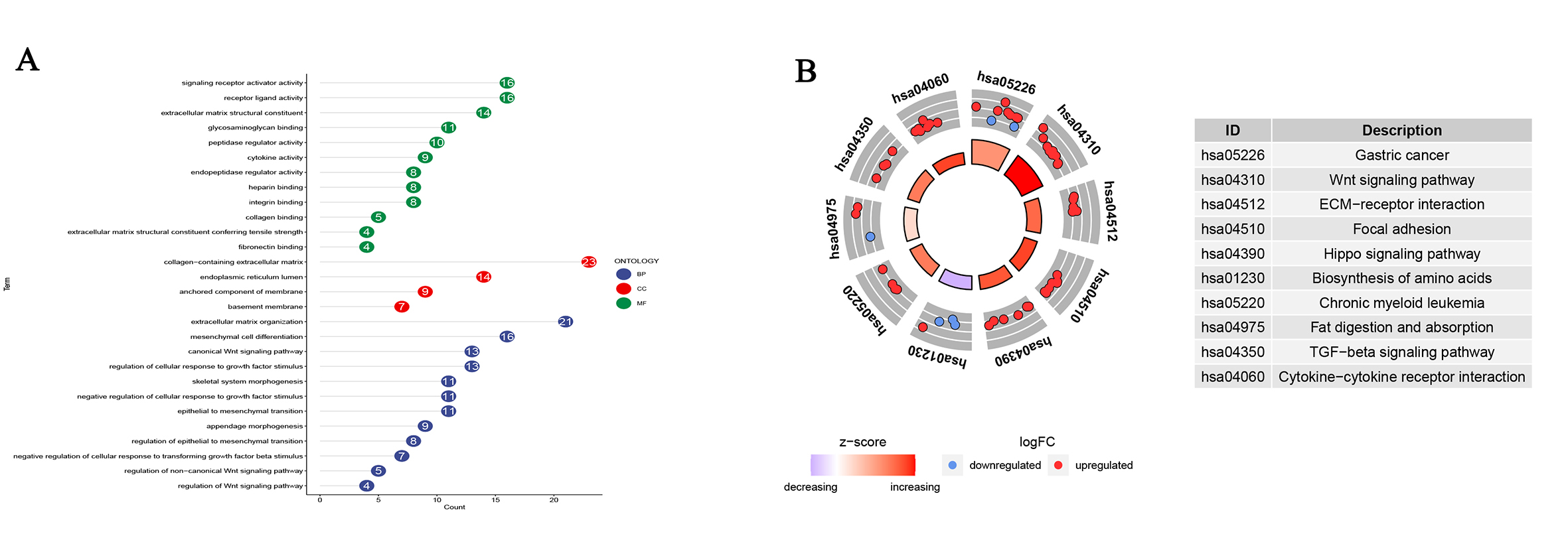

Supplement: Supplementary Figure 2 — Biological functions of differentially expressed genes (DEGs) in high- and low-risk groups. (A) Gene Ontology and (B) Kyoto Encyclopedia of Genes and Genomes analyses of DEGs in high- and low-risk groups. [file Image_2.jpeg]
